# A Young Male Presenting With Chest Pain, Elevated Troponin Levels, and a Clinical Dilemma: A Case Report

**DOI:** 10.7759/cureus.50391

**Published:** 2023-12-12

**Authors:** Ayobami B Omodara, Olusegun Areo, Joanita Kintu, Ahmed A Ziada, Mia Thornton

**Affiliations:** 1 Internal Medicine, Manchester University NHS Foundation Trust, Manchester, GBR; 2 Cardiology, Tameside and Glossop Integrated Trust, Manchester, GBR; 3 Hematology, The Christie NHS Foundation Trust, Manchester, GBR; 4 Internal Medicine, Manchester University Hospitals NHS Foundation Trust, Manchester, GBR; 5 General Internal Medicine, Manchester University Hospitals NHS Foundation Trust, Manchester, GBR

**Keywords:** st elevation, 12-lead ecg, cardiac chest pain, myo-pericarditis, cardiac troponin

## Abstract

Chest pain is a common presentation that may represent a wide variety of underlying etiologies ranging from mild self-limiting conditions to immediately life-threatening emergencies. The combination of “cardiac-sounding chest pain” and elevated troponin levels would raise suspicion of an acute ischemic event. An acute coronary syndrome is a diagnosis that may be straightforward; however, oftentimes, patients with elevated troponin levels and chest pain may bring about a state of diagnostic uncertainty. Alternative diagnoses to consider would be inflammatory or infectious conditions of the myocardium and pericardium. We present the case of a young gentleman in his twenties who presents with cardiac chest pain, elevated troponin, and non-specific changes on his electrocardiogram who was treated for an alternative cause of elevated troponin and chest pain, myopericarditis.

We present the case of a 24-year-old male who presented with a six-hour history of debilitating retrosternal chest pain. Initial workup showed a Troponin I level greater than 15,000 ng/L, D-Dimer greater than 1,000 mcg/L with no overt ischemic features on electrocardiogram. The patient had no high-risk features in his medical history & denied the use of recreational drugs. A formal same-day echocardiogram revealed normal biventricular systolic function and no evidence of regional wall motion abnormality (RWMA). He was eventually treated clinically for myopericarditis. A Cardiac MRI (CMR) imaging was done to confirm the diagnosis and rule out, most importantly, ischemic heart disease or any other underlying pathology. The main dilemma in this case was working out whether there was indeed peri-myocardial inflammation, or an acute coronary event (such as spontaneous coronary artery dissection) given his age and clinical history.

Patients presenting with a very high troponin level, particularly in young patient cohorts, should raise suspicion of a myocardial or pericardial inflammatory process. In addition to a thorough history and in the absence of ischemic changes on the electrocardiogram, subtle findings such as PR segment depression may point to a diagnosis of pericardial inflammation. While urgent echocardiography is useful to quickly assess ventricular function and for RWMA, CMR imaging is the Gold Standard modality of investigation to provide detailed structural information of the heart.

## Introduction

Myopericarditis typically presents in individuals between the ages of 20 and 50 years, who often have had a history of a recent viral upper respiratory tract infection or exposure to a new drug or vaccine [1]. Various reports have highlighted cases that appear to be linked to COVID-19 infection and/or the COVID-19 vaccines [2,3]. At present, we still come across patients in cardiology with post-COVID-19 myopericarditis syndrome, presenting with heart failure and cardiac remodeling on structural imaging [4]. Myopericarditis can present with symptoms of pericarditis, myocarditis, or a combination of both. These can vary from nonspecific symptoms such as fever and muscle tenderness, to more severe and disabling chest pain, difficulty in breathing, and extreme lethargy. Viral infections, as noted above, are the most common causes of myopericarditis in developed countries. These patients usually present with nonspecific flu-like symptoms of upper respiratory or gastrointestinal tract infection. In this case, our patient had severe chest pain that presented, albeit with a low index of suspicion, similar to a myocardial infarction. Typical electrocardiogram (ECG) features of widespread ST-segment elevation with or without concomitant PR segment depression are not always present. However, when these changes are observed, it further strengthens the suspicion of pericardial inflammation. On the other hand, myocarditis may simply appear as non-specific ECG abnormalities. The management of myopericarditis generally depends on the extent of cardiac involvement. The extent of involvement can be characterized by cardiac imaging and the mainstay in treatment is anti-inflammatory medications [1].

## Case presentation

A 24-year-old gentleman presented to the hospital with severe retrosternal chest pain which developed whilst asleep and radiated to both of his arms. On further questioning, he described intermittent, stabbing pain which was worsened by exertion and lying down, and occasionally subsided when sat up or leaning forward. The pain was the worst he had ever felt and was associated with a mild degree of breathlessness. However, he reported no nausea or diaphoresis. This event was preceded by a prodrome of viral illness described as flu-like upper respiratory tract symptoms 10 days earlier. The patient did not smoke and reported that he normally consumed roughly five units of alcohol per week. It is important to note that the patient denied the use of any recreational drug such as stimulants and hallucinogens. There was no family history of premature coronary artery disease, dyslipidemia, or sudden cardiac death. He also had no past medical history of cardiovascular or autoimmune conditions and was non-diabetic. This patient was a university student who was not usually limited in his day-to-day activities. He had not traveled abroad recently and had taken no vaccines during that year.

On initial examination, his pulse rate was 100/min and regular, blood pressure was 118/60mmHg, temperature was 37.1^o^C, capillary refill time was less than two seconds, jugular venous pulse was not raised, and there was no peripheral edema and no calf swelling or tenderness was seen. Upon auscultation of his chest, there was mild apical rub, no murmurs, and no added breath sounds in other areas of his chest. The rest of his physical examination was unremarkable. The patient appeared euvolemic and clinically stable but had ongoing retrosternal chest pain, which was not completely responsive to treatment with nitrates or opioids. An electrocardiogram was carried out twice within an hour interval, which showed no acute or dynamic ischemic changes, apart from widespread mild ST elevation without reciprocal changes. Several blood tests were taken and a presumptive diagnosis of myopericarditis with an aim to rule out non-ST elevation acute coronary syndrome (NSTE-ACS) was made.

The treatment plan was therefore for urgent ECHO, pain relief, bed rest, and serial troponins with ECGs to be taken. The patient was initially loaded by the emergency physicians on 300mg Aspirin and 180mg Ticagrelor with 2.5mg Subcutaneous Fondaparinux, following the estimation of his bleeding risk which at that time was deemed to be low while an urgent ECHO was being awaited [5]. The main variables used to estimate the patient’s bleeding risk were age, renal function, and baseline hematocrit. The rationale behind this treatment decision was to treat for an acute coronary syndrome (ACS) while an echocardiogram and urgent cardiology review was requested. On admission to the acute coronary care unit, he had a low-risk Global Registry of Acute Coronary Events Score (GRACE) of 50 points, which meant a 0.8% probability of death from admission to six months.

The patient’s history and physical examination findings were pointing away from an ACS. A bedside echocardiogram was performed shortly afterward by a member of the cardiology team (see Investigations section). Together with serial Electrocardiograms and cardiac monitoring via telemetry, no dynamic ischemic changes were shown. Considering all these results together, as well as raised C-reactive protein (CRP) level, the cardiology team then felt more confident to treat as likely myopericarditis. Given that he was in his early twenties with a low GRACE score, the preferred line of further invasive cardiac testing was one that would mitigate the risk of undue exposure to contrast and ionizing radiation, i.e., cardiac magnetic resonance (CMR) imaging. He was started on Colchicine, high-dose non-steroidal anti-inflammatory drugs (NSAIDs), and Lansoprazole (PPI) in line with the ESC 2015 guidelines [1,6]. Medical therapy for ACS was stopped. The patient was then subsequently referred for a CMR. With respect to the diagnostic reliability of CMR in myocarditis, it carries a sensitivity of 81%, a specificity of 71%, and a diagnostic accuracy of 79% [7].

Investigations 

The initial laboratory investigations for the patient's chest pain are given in Table 1.

**Table 1:** Initial investigations and diagnostic workup done to investigate the patient's chest pain

Investigation	Result	Reference Range
Haemoglobin	142 g/L	130 - 180 g/L
Total White Cell Count	5.9 x10^9^/L	4 - 11 x10^9^/L
Platelets	164 x10^9^/L	130 - 400 x10^9^/L
ESR	21 mm/hr	<10 mm/hr (male)
C-reactive Protein	78 mg/L	0 - 8 mg/L
Sodium	138 mmol/L	133 - 146 mmol/L
Potassium	4.0 mmol/L	3.5 - 5.3 mmol/L
Urea	2.7 mmol/L	2.5 – 7.8 mmol/L
Creatinine	64 micromol/L	60 - 105 micromol/L
eGFR	>90 mL/min/1.73m^2^	90 - 120 mL/min/1.73m^2^
Magnesium	0.91 mmol/L	0.70 - 1.0 mmol/L
INR	1.2	0.9 - 1.2
D-dimer	1084 mcg/L	0 - 500 mcg/L
COVID-19 Rapid Antigen Test	Negative	
COVID-19 PCR	Negative	

The cardiac biomarkers used to investigate the patient's chest pain are given in Table 2.

**Table 2:** Trend of cardiac biomarkers used to investigate patient's chest pain

Investigation	Result	Reference Range
Troponin I (0 hours)	13,564 ng/L	<19.8 ng/L
Troponin I (3 hours)	15,129 ng/L	<19.8 ng/L
Troponin I (>24 hours)	12,840 ng/L	<19.8 ng/L
NTproBNP	454 pg/mL	<400 pg/mL

Electrocardiography Findings

**Figure 1:**
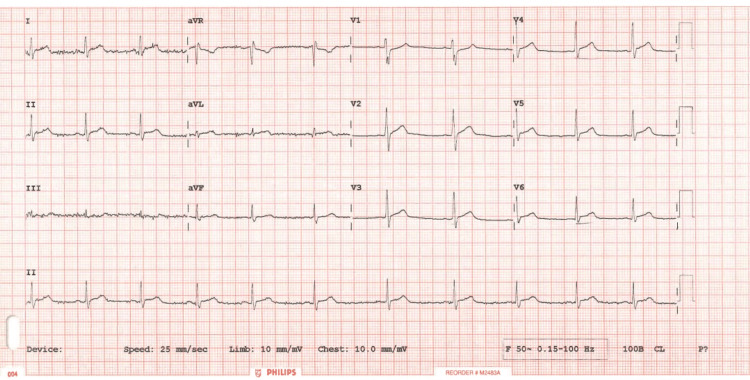
12 lead Electrocardiogram on admission showing widespread ST elevation and relative PR depression most prominent in Leads I, II, aVL, V2-V6. A “reciprocal phenomenon” is also observed in aVR and V1. Note here that the reciprocity is dissimilar to that observed in acute coronary syndrome.

Sinus rhythm with widespread Concave anterolateral ST elevation (no reciprocal changes noted to suggest an MI). There was some degree of PR segment depression. Features all pointing towards pericardial involvement with high troponin levels suggestive of extensive myocardial injury (Figure 1). See the first ECG taken in the Accident & Emergency Department.

**Figure 2:**
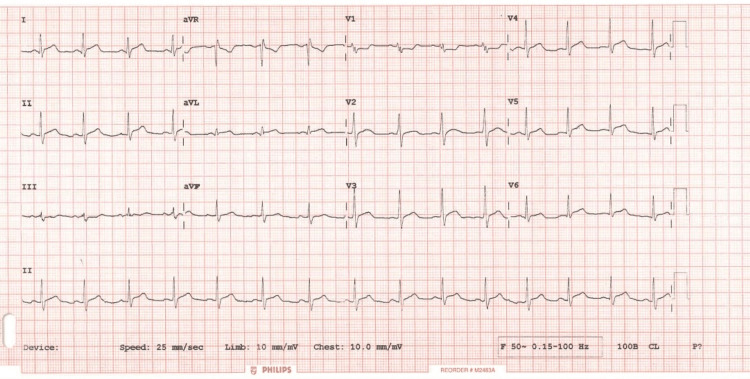
12 lead Electrocardiogram one hour later showing similar changes as the ECG done on admission. No significant changes were observed; however, ST segment depression was more pronounced.

The second ECG taken as part of a set of serial ECGs to look out for dynamic ischemic changes (Figure 2).

Imaging

His chest x-ray showed a normal cardiothoracic ratio and no abnormality was detected. ECHO revealed normal biventricular systolic function with an ejection fraction greater than 60%. No significant valvular abnormalities were detected. No regional wall motion abnormality (RWMA) was found. Trivial anterior pericardial effusion noted. 

**Figure 3:**
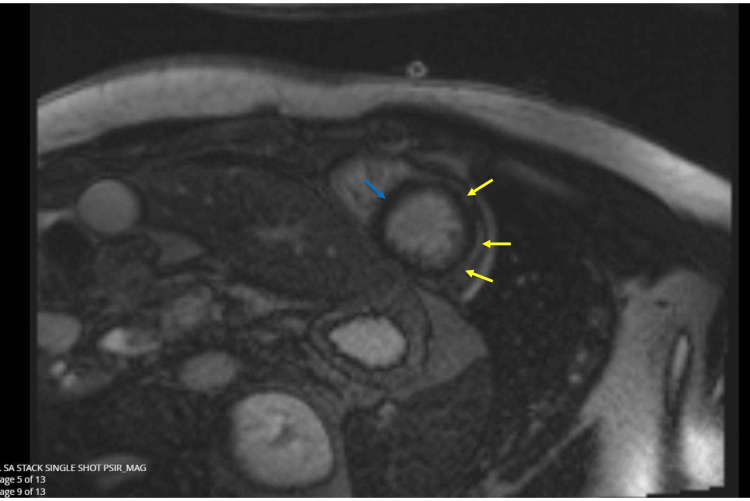
In the late phase, following gadolinium contrast, there is subepicardial late gadolinium enhancement at the apical myocardium, midventricular anterolateral wall and midventricular inferolateral wall (see yellow arrows) with sparing of the apical septum (blue arrows).

**Figure 4:**
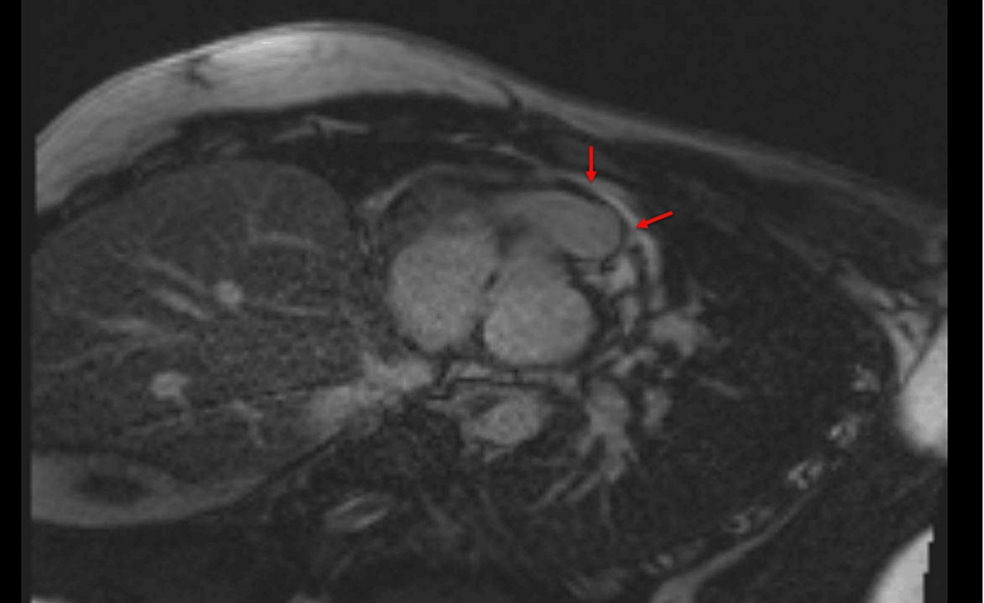
Cardiac Magnetic Resonance imaging showing mid-wall myocardial enhancement at the basal anteroseptal wall and basal anterolateral wall (see red arrows)

CMR revealed normal bi-atrial size with a left atrium measuring 9cm/m^2^ (7-15cm/m^2^) and a right atrium measuring 9cm/m^2^ (8-16cm/m^2^). The left ventricle was of normal diameter and volume. There was, however, subtle hypokinesia of the midventricular inferolateral wall. The left ventricle was of normal wall thickness. His Right Ventricle was normal in size and volume with normal longitudinal function with a tricuspid annular plane systolic excursion (TAPSE) of 20mm and normal wall thickness. Left ventricular ejection fraction was 61% (57%-74%), stroke volume (SV) 112mL/49mL/m^2^ (44-68mL/m^2^) and mass was 126/55g/m^2^. Right Ventricular ejection fraction was 60% (48%-74%) and SV was 113mL/49mL/m2 (40-72mL/m^2^). There were no valvular abnormalities, and the aortic configuration was normal. No pericardial effusion was detected on CMR. Normal nulling sequence. In the late phase following gadolinium contrast, there was subepicardial late gadolinium enhancement at the apical myocardium, midventricular anterolateral wall, and midventricular inferolateral wall with sparing of the apical septum (Figure 3). There was mid-wall myocardial enhancement at the basal anteroseptal wall and basal anterolateral wall (Figure 4). T2 mapping values were also mildly elevated at the subepicardial region of corresponding myocardial segments (up to 48 ms), signifying probably resolving myocardial edema. The overall impression was that of resolving myopericarditis with normal biventricular size and systolic function. Of note, CMR excluded ischemic heart disease in this patient.

Differential diagnosis

The first responders (ambulance crew) were concerned about the patient’s symptoms which with the associated ECG changes, could suggest ST elevation Myocardial Infarction. This was a reasonable differential to consider, however, there were certain features that deemed it unlikely. Other features that made an ACS less likely in this young man included: age, medical and social history, pattern of chest pain evolution, and Electrocardiographic changes. While spontaneous coronary artery dissection (SCAD) could present as ACS in young people, it tends to occur more commonly in females or those with a history of short-burst heavy lifting or physical exertion without sufficient prior training [8]. This was not consistent with our patient’s history. These features combined put this young man in a low-risk category for ACS.

Moreover, we ruled out post-COVID-vaccine reactions, pneumonia, or pneumothorax based on the patient's history, negative COVID-19 test, and normal chest x-ray. The mediastinum and cardiac silhouette were normal (cardio-thoracic ratio: 0.48). Other potential causes, such as Teitz syndrome, musculoskeletal chest wall injuries, pleurisy/pleuritis, and a dissecting thoracic aneurysm, were eliminated due to the absence of physical signs, supported by normal echocardiogram, ECG, and blood pressure results in both arms [9]. The patient showed no chest wall tenderness on palpation and had no syncope or pre-syncopal episodes before admission. Additionally, pulmonary embolism was an unlikely diagnosis as the patient had no previous personal or family history of venous thromboembolism, no history of malignancy, no calf swelling, or prolonged immobilization such as a long-haul flight or hospital admission. In addition, there were no echocardiographic features of pulmonary embolism such as raised RV pressure, strain pattern, or right ventricular dilatation. He had a low Well's score for pulmonary embolism of 1.5 (PE unlikely) and had no history of previous venous thromboembolism.

Although several cardiologists nowadays would still opt for invasive coronary imaging, a cardiology Multi-Disciplinary Team meeting was held, and the decision was made not to proceed with an invasive coronary angiography, rather, CMR imaging was deemed sufficient. According to the ESC guidelines, the important features to look out for when patients present with symptoms suggestive of pericarditis include evidence of myocardial involvement, pericardial effusion with or without tamponade, and malignant arrhythmias. These features will generally determine whether a patient should be treated in an outpatient setting or admitted for inpatient monitoring.

Treatment 

The ESC maintains that the first line therapy in uncomplicated cases of pericarditis, depending on the extent of myocardial involvement, should be a combination of high dose Aspirin (or other NSAIDs) and Colchicine. However, steroids can be used as an alternative therapy where there is documented evidence of intolerance or allergy to Aspirin/NSAIDs and Colchicine [10]. Steroids could also be considered in more complicated cases such as with concurrent myocardial necrosis. It is important to note that care should be taken with the use of steroids in the immunosuppressed. In such cases where there is strong evidence of myocarditis, other medications such as beta blockers and ACE inhibitors should be considered depending on the patient’s left ventricular function and risks of cardiac remodeling. In our patient’s case, findings from an ECHO, ECG, and CMR provided enough evidence to exclude myocardial damage or involvement.

Colchicine can cause disabling side effects in some patients such as severe diarrhea and vomiting. It is also known to have a narrow therapeutic index and hence needs close monitoring. As a result, a short course is usually preferred, ideally between one and three months. Steroids, when prescribed, require slow tapering and have numerous side effects including anxiety, gastrointestinal discomfort, hypertension, and even the risk of peptic ulcers, osteoporosis, Cushing’s syndrome, etc. Our patient was given a high dose of Naproxen and a short course of Colchicine, which was well tolerated.

According to several guidelines across the world, it is unanimously agreed that patients confirmed to have peri-myocarditis, particularly those with significant myocardial involvement, should avoid exercise during the first three to six months [11]. This may pose a significant challenge in some cases, as a considerable number of patients affected by this condition are young and otherwise fit, some of whom are elite athletes. Peri-myocarditis is one of the few cardiac diseases where exercise has been proven to be counterproductive and should be avoided in the initial/acute inflammatory phase [11,12]. For instance, a study by Preito-Hinojosa et al., which investigated the immune system activity of athletes, found that naïve T-cell numbers and thymic output were severely reduced in elite athletes. As a result, their immune systems mirrored those of a much older patient population. The impact of these changes on myocarditis risk has not yet been explored [13].

Outcome and follow-up

The patient was advised to rest for a further month before getting back to intense physical exertion. One month was recommended due to the rather benign findings on his ECHO and CMR. Given that this patient is young, we envisioned and recognized the likely difficulty in compliance that that could pose. We arranged a follow-up clinic three months after his CMR. The patient was discharged home with Naproxen 500mg three times daily for three months, and Colchicine 500 mcg twice a day for a month with monitoring of side effects such as diarrhea. ACS treatment was discontinued. As highlighted earlier, colchicine has a narrow therapeutic window and a risk of serious and fatal toxicity in overdose [14]. Therefore, patients are generally warned to report side effects promptly. He was also prescribed omeprazole 20mg once a day for the same period. His general practitioner (GP) was sent out a letter to follow up with the patient’s medication prescription and to ensure stopping them when appropriate.

Our patient remained in a stable condition and had no reason to re-attend hospital with this condition. Outpatient CMR results are attached to this report. Furthermore, to continue follow-up, the patient will have an echo in one year. The echocardiogram results will determine if any further investigations are required.

## Discussion

In our case report, this gentleman was brought in with excruciating retrosternal chest pain which was not alleviated despite opioid and GTN use. Important clues to an accurate diagnosis here include the patient’s demographics, pattern of pain, lack of use of stimulant drugs (e.g. cocaine), an apical friction rub on auscultation, and a preceding viral prodrome of flu-like illness. It is important to note, however, that it is not impossible for young patients to present with ACS. More common causes of ACS in the younger population include SCAD from over-exertion, arteritis from autoimmune causes or vasculitis, or congenital heart disease such as abnormal coronary artery anatomy and premature coronary artery disease [15]. These were less likely given the absence of any regional wall motion abnormalities on the echocardiogram performed by one of the cardiologists.

In this patient, the significantly raised initial troponin levels and CRP were likely a result of myocardial inflammation rather than ACS, although the latter could not be fully excluded based only on these troponin levels. In such a clinical dilemma, we later decided it was safer to treat it as peri/myocarditis following a full-loading therapy for an ACS (NSTE-ACS). This was a decision made by a cardiology Multi-Disciplinary Team meeting where the patient’s history, ECG, and findings from imaging were discussed. On further ECG analysis, a raised J-point with a concave ST segment elevation and relative PR segment depression in anterolateral leads might be misleading and lead some clinicians to consider an ACS. Particularly important is the absence of reciprocal ischemic changes in other leads which in most cases, points away from an ACS.

According to ESC guidelines, cases of pericarditis with suspected associated myocarditis should be managed and investigated according to the patient’s clinical symptoms, cardiac risk factors, and demographics. Where applicable, Coronary Angiography could be performed (Class1C evidence) and CMR is recommended for confirmation of myocardial involvement (Class IC). It further recommends hospitalization for diagnosis and monitoring in patients with myocardial involvement and advises rest and avoidance of physical activity beyond normal sedentary activities in non-athletes and athletes with myopericarditis for a duration of six months (Class IC evidence) [10].

Nevertheless, when faced with such an emergency in an acute setting, a bedside ECHO by trained professionals can provide reassurance in the absence of significant RWMA and/or significant pericardial collection. Such patients should ideally be reviewed by the cardiology team as a matter of urgency, particularly in more unstable patients with high-risk ECHO and/or ECG features. In situations where there is any diagnostic uncertainty, it should generally be safe to cover for both Myopericarditis and ACS until further imaging is made to confirm the diagnosis.

## Conclusions

A thorough history is crucial in formulating a sensible preliminary diagnosis that can be supported or refuted by subsequent investigations. An initial very high troponin level, e.g., in tens of thousands in a very young patient should always trigger questions about a myocardial +/- pericardial inflammatory origin rather than focusing solely on vascular causes. However, the exclusion of an acute coronary event remains an important part of practice in patients presenting in this manner. ECG changes such as PR segment depression are subtle findings that may suggest pericardial inflammation in the first instance and should thus be taken into account in the context of chest pain without typical ischemic changes. While urgent echocardiography is useful to quickly assess ventricular function and for RWMA, CMR imaging is the Gold Standard modality of investigation to provide detailed structural information of the heart. Unlike most other cardiovascular conditions, patients diagnosed with Myopericarditis are advised to avoid excessive physical exertion for several months. This is one of the few cardiac conditions in which exercising could worsen the patient's outcome.
